# Editorial: Personalized nutrition therapy in critical illness and convalescence: moving beyond one-size-fits-all to phenotyping and endotyping

**DOI:** 10.1097/MCC.0000000000001060

**Published:** 2023-07-06

**Authors:** Arthur R.H. van Zanten

**Affiliations:** Department of Intensive Care Medicine & Research, Gelderse Vallei Hospital, Ede, The Netherlands

## INTRODUCTION

Recent research has highlighted the limitations of traditional feeding guidelines for patients, which fail to consider individual requirements, body composition, and metabolic stress. However, personalized nutrition therapy has emerged as a promising approach to improve outcomes and prevent adverse effects. Phenotyping involves considering patient-specific data and body composition to determine the ideal macronutrients and micronutrients during various stages of illness and recovery. By employing endotyping, which identifies distinct disease-related mechanisms and metabolic biomarkers, tailored nutritional plans can be developed. This edition of Current Opinion in Critical Care focuses on the latest insights into personalized nutrition therapy.

## INDIVIDUALISED ENERGY STRATEGY

Energy provision in nutritional support is crucial, but the outdated approach of using total body weight and predictive equations for dosing should be discarded [1]. Phenotyping, which considers patient characteristics like BMI and nonintentional calorie intake, is essential [2–3]. Patients with higher BMIs may need increased energy intake to maintain metabolic balance, while those receiving nonintentional calories (e.g., propofol, citrate, or dextrose-containing fluids) may require lower energy intake to prevent overfeeding [4]. Additionally, energy requirements can significantly differ throughout the different phases of critical illness [5].

Endotyping involves measuring variations in energy expenditure using indirect calorimetry (IC), recently recommended by international nutrition guidelines, and assessing endogenous energy production to determine individual energy requirements. Although relevant, simple bedside determination of endogenous energy production is not possible [6].

Early full-dose feeding is associated with adverse outcomes as endogenous energy production then is not suppressed by nutrition therapy and administration of insulin [7]. Therefore, full feeding in the early phase may induce overfeeding. Considering whether the provided energy promotes anabolic processes or induces insulin resistance and hyperglycaemia is crucial [8]. Optimal glycaemic targets are addressed by Gunst *et al.* in this journal [9]. Currently, we lack biomarkers to guide energy intake in critical illness and recovery phases. However, a high respiratory quotient (RQ), elevated insulin needs, and exceeding 110% of REE based on indirect calorimetry measurements may indicate metabolic intolerance (endotyping) [10–13]. In a recent meta-analysis on individualized energy strategies [14], a 23% decrease in mortality was observed with personalized energy provision based on indirect calorimetry (IC). However, concerns were raised regarding study size, inconsistent mortality data, and the lack of mortality reduction in individual studies or impact on other patient outcomes [15]. Nevertheless, experts and guidelines endorse the use of IC-based strategies in guiding nutrition therapy [1,16].

Composition and routes of administration of energy sources are also relevant. Typically, parenteral nutrition contains all three macronutrients. Haines *et al.*, in this journal edition, address that with similar energy doses better outcomes are observed when soy-based lipids are switched to fish-oil-based lipid emulsions [17]. Mitochondrial dysfunction may induce an intracellular energy crisis [18]. Alternative energy sources then should be considered. The role of ketones with anticatabolic properties as promising alternatives is addressed in this edition by Watson *et al*. [19].

Personalized energy strategies, incorporating phenotyping and endotyping, may surpass the traditional one-size-fits-all approach for critically ill patients. However, more research is necessary to establish standardized methods for determining individual energy needs and evaluating the long-term effects of personalized energy provision on clinical outcomes.

## INDIVIDUALISED PROTEIN STRATEGY

Adequate protein intake is crucial for muscle mass, immune function, and wound healing in critically ill patients. However, a one-size-fits-all approach to protein dosing based on total body weight may not be effective. Individualized protein dosing based on phenotyping and endotyping is recommended. Phenotyping involves identifying patient characteristics that may affect protein requirements, such as body mass index (BMI), sex, lean body mass (LBM), and age. Studies have shown that men generally require higher protein intake than women due to their larger body size and higher LBM [20]. However, in patients of the same sex and total body weight, variations in LBM can result in potential protein overfeeding or underfeeding. Elderly patients and those with lower BMIs may also have higher protein requirements to prevent muscle wasting. Increasing protein dose has been associated with reduced but also enhanced muscle mass loss [21].

Early high protein intake may worsen clinical outcomes, as has been shown in an observational study and posthoc analyses of randomized controlled trials (RCTs) [22–24]. Recently, our group also found associations with increased mortality of high protein intake during the emergence of refeeding hypophosphatemia [25]. Therefore, guidelines have recommended gradual progression to the protein intake target [1]. For this reason, in our ICU, we apply daily steps of 25% increase both for proteins and energy [26].

A recent meta-analysis found no evidence of benefits on clinical outcomes, including mortality and length of stay, when increasing protein intake by approximately 0.5 g/kg per day during critical illness [27]. This was confirmed in the EFFORT trial by Heyland *et al.* comparing an achieved protein target of 1.6 vs. 0.9 g/kg per day that showed no differences in outcomes. Moreover, in the subgroup of patients with acute kidney injury and high SOFA scores, increased mortality was observed [28]. More studies on high protein interventions in ICU are ongoing such as the PRECISE trial (NCT04633421) and the Target protein trial (ACTRN12621001484831).

Metabolic tolerance of amino acids varies among patients and changes during critical illness and convalescence. Endotyping, using intracellular biomarkers, can identify specific protein needs when phenotyping is insufficient. In ICU patients, despite similar protein digestion, absorption and plasma amino acid levels compared to healthy controls, severe anabolic resistance leads to significantly lower muscle protein synthesis, indicating the impact of critical illness on protein metabolism [29]. The mechanisms involved in these processes are not fully understood, but potential factors include mitochondrial dysfunction, intracellular energy depletion, proteostatic phenotype induction, and disruption of cellular metabolic processes through autophagy suppression [30–31]. The role of autophagy in critical illness is discussed in detail in a comprehensive overview by Vanhorebeek *et al.* in this journal [32].

Proteins and amino acids not used for muscle protein synthesis can be metabolized in various ways. Amino acids can be converted to glucose through gluconeogenesis in the liver. Excess amino acids are metabolized in the liver, producing ammonia, which is then converted to urea for safe excretion. The urea–creatinine-ratio (UCR) may be a useful biomarker for monitoring whether ingested proteins are utilized for muscle protein synthesis, oxidized for energy, or metabolized to urea. High UCR levels in patients from the REDOXS trial were linked to adverse clinical outcomes, particularly increased mortality with high-dose glutamine supplementation [33]. Incorporating UCR into the analysis revealed that the association between glutamine and increased mortality disappeared, indicating that it was the higher amino acid dosage, as indicated by elevated UCR levels, rather than glutamine itself, that was metabolically intolerable. These findings may also help explain the adverse effects of high-protein intake in patients with high SOFA scores, early-phase critical illness, and AKI patients, as inflammation is the primary driver of catabolic responses and anabolic resistance.

**FIGURE 1:**
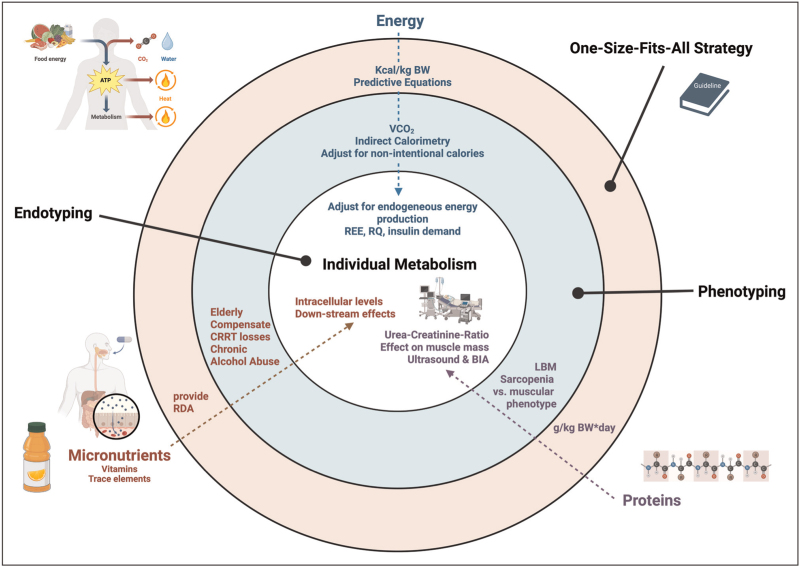
Moving from one-size-fits-all strategies in nutrition therapy to phenotyping and endotyping critically ill patients to meet individual metabolic demands in all phases of disease and convalescence. Instead of relying on general population-based averages for energy, protein, and micronutrient intake, individualized nutrition strategies should be tailored to patients based on specific phenotypic factors such as sex, age, body composition, and treatments. Additionally, endotyping can be used to assess the metabolic condition of the patient and predict how nutrients will be utilized, either for beneficial metabolic processes or potentially harmful effects. Personalized nutrition plans based on individualized daily demands and endotyping potentially can improve patient outcomes. ATP, adenosine triphosphate; BIA, bioelectrical impedance assessment; BW, body weight; CO_2_, carbon dioxide; CRRT, continuous renal replacement therapy; g, grams; kcal, kilocalories; kg, kilograms; LBM, lean body mass; RDA, recommended daily allowances; REE, resting energy expenditure; RQ, respiratory quotient; VCO_2_, production of CO_2_ per minute.

Personalized protein dosing based on phenotyping and endotyping could help optimize nitrogen balance, prevent muscle wasting, and improve clinical outcomes in critically ill patients while avoiding the harmful effects of excessive protein intake. Additionally, monitoring the loss of muscle mass via muscle ultrasound or BIA during ICU stay may be a helpful tool for following up with individual patients at risk (Fig. 1).

## INDIVIDUALISED MICRONUTRIENT STRATEGY

Micronutrients, such as vitamins and trace elements, play a critical role in maintaining an individual's overall health, particularly during times of stress and illness. However, feeding ICU patients has traditionally relied on a one-size-fits-all approach, using recommended daily allowances as a guideline [34]. Instead, a personalized approach based on phenotyping and endotyping may be more appropriate.

Phenotyping involves identifying patient characteristics, such as age, sex, and medical history, that may affect micronutrient requirements. For example, elderly patients may have reduced absorption and utilization of certain vitamins like vitamin D, while those with renal impairment may experience increased losses of micronutrients during continuous renal replacement therapy. To prevent micronutrient deficiencies, early multisupplementation with low doses of various micronutrients can be considered for specific phenotypes. This is especially relevant when enteral feeding administration is progressed gradually over 4–5 days and the target of 1500 kcal or 1500 ml of tube feeding, with sufficient micronutrients to meet the recommended daily allowance (RDA), is not achieved.

Endotyping involves measuring intracellular biomarkers or downstream metabolic activity to identify specific micronutrient deficiencies not reflected in serum levels.

Micronutrients in critical illness are excellently addressed by Koekkoek and Berger in this journal [35]. In critical illness, ionized calcium levels are typically lower. Melchers and Van Zanten discuss in this issue whether this indicates adaptation or necessitates calcium supplementation [36].

Radke *et al.*[37] provide the latest insights on high-dose vitamin C supplementation in this journal. Although some studies indicate potential benefits of high-dose micronutrient supplementation for critically ill patients, it is crucial to recognize the adverse effects of excessive doses. Currently, high-dose supplementation is not recommended for ICU patients. In summary, a personalized approach to micronutrient supplementation based on phenotyping and endotyping may be more effective in optimizing the nutritional status of ICU patients. However, further research is needed to develop this approach.

## OVERCOMING BARRIERS TO ACHIEVING NUTRITIONAL TARGETS IN CRITICAL ILLNESS

Nutrition therapy in critical illness faces barriers hindering its effectiveness. Viner Smith *et al.* note low intake in patients receiving nasal high-flow oxygen therapy or noninvasive ventilation [38]. Gastrointestinal (GI) dysfunction further hampers nutrition therapy by affecting GI motility, nutrient digestion and absorption, and gut metabolism. To address these challenges, researchers have explored strategies such as energy-dense and elemental formulas, prokinetics, intermittent feeding, and postpyloric feeding, with varying success. Van Gassel *et al.*[39] provide a comprehensive exploration of this topic. In the post-ICU phase, De Waele *et al.*[40] present updated insights on feeding practices and strategies to improve macronutrient intake during convalescence.

## CONCLUSION

Personalized nutrition therapy is essential for better outcomes in critically ill patients, necessitating an individualized approach based on phenotyping and endotyping. Energy strategies tailored to the patient's phenotype and guided by indirect calorimetry can be more effective than traditional weight-based approaches. Likewise, protein dosing should be personalized to prevent underfeeding or overfeeding, considering metabolic tolerance. Biomarkers like the UCR show promise in assessing protein metabolism. Cellular homeostasis during critical illness requires adequate vitamins and trace elements. However, the traditional method of providing micronutrients based on recommended daily allowances may not suffice, calling for a personalized approach based on phenotyping and endotyping. Identifying specific deficiencies through intracellular and downstream biomarkers is crucial. Nonetheless, implementing this complex personalized approach poses challenges. High-dose micronutrient supplementation is not recommended due to potential adverse effects and lack of proven benefits.

This special edition of Current Opinion in Critical Care offers valuable insights for effective and individualized nutrition therapy.

## Acknowledgements

*I like to express my sincere gratitude to all the contributors and the editor-in-chief, who have dedicated their time and effort to share their insights and expertise and have made this special edition possible.*

### Financial support and sponsorship

*None.*

### Conflicts of interest

*Professor Van Zanten reported receiving honoraria for advisory board meetings, lectures, research, and travel expenses from Abbott, AOP Pharma, Baxter, Cardinal Health, Danone-Nutricia, Dim-3, Fresenius Kabi, GE Healthcare, Medcaptain, Mermaid, Nestlé, PAION, Lyric, and Rousselot.*

## REFERENCES AND RECOMMENDED READING

Papers of particular interest, published within the annual period of review, have been highlighted as:

▪ of special interest
▪▪ of outstanding interest
